# Effect of erosive media on microhardness and fracture toughness of CAD-CAM dental materials

**DOI:** 10.1186/s12903-022-02230-1

**Published:** 2022-05-19

**Authors:** Alaaeldin Elraggal, Rania Afifi, Islam Abdelraheem

**Affiliations:** Conservative Dentistry Department, Faculty of Dentistry, Alexandria University, Champollion street, Alexandria, Egypt

**Keywords:** CAD-CAM, Monolithic zirconia, Lithium disilicate, Resin composite, Hybrid ceramic, Fracture toughness, Microhardness

## Abstract

**Background:**

Erosive acids might create surface flaws and deteriorate the mechanical properties of CAD-CAM materials. This invitro study aimed to investigate the effect of simulated gastric HCl and extrinsic erosive acids on surface microhardness and fracture toughness of CAD-CAM materials.

**Methods:**

400 bar-shaped specimens (17×4×2 mm^3^) were prepared from 4 different CAD-CAM dental materials (n = 100/group); monolithic zirconia (Ceramill Zolid HT+, Amanngirbach, Austria), lithium disilicate ceramic (IPS e.max CAD, Ivoclar Vivadent, Liechtenstein), nanohybrid resin composite (Grandio Blocs, VOCO) and polymer-infiltrated glass network (Vita Enamic, VITA Zahnfabrik). Specimens from each material type were further subdivided into 5 groups (n = 20) according to the erosive media applied (simulated gastric HCl, white wine, Coca-Cola®, orange juice, and artificial saliva that served as a control). Specimens were immersed for 24 h in an incubator at 37 ℃, then ultrasonically cleaned in distilled water and air-dried. Half of the specimens were tested for Vickers microhardness (VHN) at parameters of 500 gf for 10 s, while the rest of the specimens underwent 3-point flexure till fracture. Fractured surfaces were examined under a scanning electron microscope (SEM) for fracture toughness (K_IC_) calculation using the quantitative fractographic analysis method. Data collected were statistically analyzed using two-way analysis of variance (α = 0.05) after verification of data normality and homogeneity of variances.

**Results:**

Erosive media created surface flaws that lowered the surface microhardness of the material and initiated the fracture pattern under different loads according to material type. The material type was a more predominant factor than erosive media that affected either the microhardness or the fracture toughness of CAD-CAM dental materials. The highest VHN and K_IC_ values were found among Ceramill Zolid HT+ groups followed by IPS e.max CAD and Grandio Blocs regardless of the erosive media employed. Erosive media significantly reduced the VHN and K_IC_ in Vita Enamic specimens compared to the rest of the material types.

**Conclusion:**

All CAD-CAM materials used, except Vita Enamic, showed high resistance against the erosive acids indicating higher longevity of the material in patients frequently exposed to either extrinsic or intrinsic acid.

## Background

Dental erosion can be defined as an irreversible loss of tooth enamel and/or dentin due to frequent exposure to erosive acids of non-bacterial origin [1]. These acids might originate internally, as a pathological feature of certain systemic diseases such as gastroesophageal reflux disease (GERD) or bulimia nervosa [2, 3]. Acid-containing diets such as fizzy drinks, citrus fruits, and white wine represent an external source of acids the frequency of which might eventually lead to dental erosion [4]. In clinical practice, patients are asymptomatic to the early tooth hard tissue loss from dental erosion [5]. However, neglected cases might lead to a substantial tooth loss leading to complaints of dentin hypersensitivity, loss of vertical dimensions, loss of teeth anatomy, contour, and function [4, 6, 7]. Dental erosion is more frequent in the age range of 9–19 years old with a prevalence of 39% around the world [5]. Management of dental erosion has gained more attention in dental practice due to the alarming rise in the variety of acid-containing food and beverages that are consumed heavily by the population [8].

Restorative treatment of dental erosion will depend on the stage at which patients seek dental advice [9]. Early manifestations, where tooth loss is still within the enamel surface without loss of contour might be treated by controlling the underlying cause, fluoride varnish application, diet advice, and simple resin composite restorations for individually-affected teeth [9]. However, in late stages, where teeth anatomy, contour, and height were significantly lost with symptomatic complaints, can be treated by full mouth rehabilitation [10].

The revolutionary innovations in CAD-CAM dental materials such as resin composites, glass–ceramics and zirconia have been accompanied by a significant increase in their application in restorative and esthetic dentistry owing to their more precise fit, high mechanical properties, and ultimate reproduction of the natural appearing of teeth [11–14]. The implementation of CAD-CAM technology has extended their use from chair-side dental restorations of decayed or fractured teeth to their potential use as dental implant, implant abutments and full arch fixed bridges [15]. Nevertheless, the frequent exposure of CAD-CAM materials to extrinsic or intrinsic acids might lead to surface deterioration and flaws that may eventually doubt the fracture toughness and hence, the durability of the restoration [6, 16]. The presence of surface flaws might act as crack initiators which, under occlusal loads, might propagate leading to a catastrophic failure [17, 18]. The effect of erosive media on the surface roughness, microhardness, and optical properties has been reported in the literature for limited types of CAD-CAM materials [3, 6, 19, 20]. To the authors' knowledge, the effect of erosive media on fracture toughness of CAD-CAM materials has never been reported. Further, there is scarce data detailing the effect of erosive media on the microhardness of a wider variety of CAD-CAM materials including monolithic zirconia, lithium dislicate, hybrid ceramics, and resin composite. Therefore, the current study aims to evaluate the effect of different erosive media including simulated gastric HCl, white wine, orange juice, and Coca-Cola® on surface microhardness and fracture toughness of monolithic zirconia, lithium disilicate ceramic, Polymer-Infiltrated Ceramic Network (PICN), and nanohybrid resin composite. Quantitative fractographic analysis was also conducted to locate the origin and the pattern of fracture in different CAD-CAM materials. The study was guided by the null hypothesis that different erosive media would not affect the microhardness and fracture toughness of studied CAD-CAM materials.

## Materials and methods

### Specimens’ preparation

A total of 400 bar-shaped specimens were obtained by sectioning 4 types of CAD-CAM materials: high-translucent zirconia (Ceramill Zolid HT+, Amanngirbach, Austria) (HZ), lithium disilicate glass–ceramic (IPS e.max CAD; Ivoclar Vivadent, Liechtenstein) (EC), PICN (Vita Enamic; Vita Zahnfabrik) (VE), and nano-hybrid resin composite (Grandio blocks; Voco, Cuxhaven, Germany) (RC). All CAD-CAM blocks, except zirconia, were sectioned into targeted dimensions of (17 × 4 × 2 mm^3^) using a precision diamond cutting machine (IsoMet 4000 Buehler, Germany). Zirconia blocks were sectioned at higher dimensions of (20.9 × 4.9 ×  2.5 mm^3^) to compensate for a later volumetric sintering shrinkage of approximately 20% to reach similar dimensions of other CAD-CAM specimens. Zirconia bars were then sintered at 1450 °C for 9 h in a Zirconia sintering furnace (Ceramill Therm 3, Amann girrbach, Austria). In the firing cycle, the temperature increased gradually from room temperature to sintering temperature of 1450 °C in 30 min. Sintering temperature was retained by the furnace for 9 h after which it started to descend in a cooling phase till 1140 °C in 8 min. The furnace then opened gradually while the temperature kept descending till 540 °C in 14 min, which represented the end of the firing cycle. Specimens were then taken out of the furnace and left to cool down to room temperature. IPS e.max CAD specimens underwent crystallization at 840 °C in a porcelain firing furnace (Programat P310, Ivoclar Vivadent) following the manufacturer’s recommendations. In the sintering cycle, the temperature was raised from room temperature to 830 °C at a rate of 90 °C/min. The temperature was then further increased to 840 at a rate of 30 °C/min and retained at this degree for 7 min before automatic cooling down to 710 °C representing the end of the sintering cycle. Specimens were then taken out and left to cool till reaching the room temperature. The dimensions and perpendicularity of faces of the tested bars were checked and verified by measuring the length, width and thickness of specimens at 4 separate points placed equally from each other along the bar using a digital caliper (QuantuMike, Mitutoyo). Specimens that showed similar measurement outcomes at the four different points were included in the study. All bars were then polished with metallographic silicon carbide paper at a sequence of (600- 800- 1000- 1200-grit) and underwent ultrasonic cleaning in distilled water for 10 min.

### Erosive media

One hundred bars from each material were then assigned, according to exposure to different media, into five groups of 20. The bars were inserted into one of 5 of the following media:

1. Lab-prepared simulated gastric acid (0.113 wt% HCl in deionized water) [6].
2. Fresh orange juice (obtained from freshly squeezed orange fruit). The main acid components in orange juices are citric, malic and ascorbic acids.
3. 12.5% alcohol-containing white wine (Omar Alkhayam, Gianaclis Vineyards company for beverages, Egypt). White wines contain erosive acids such as lactic acid, succinic acid, acetic acid, and tartaric acids.
4. Phosphoric acid-based sugar-containing fizzy drink (Coca-Cola®).
5. Artificial saliva served as a control group. An artificial saliva was prepared according to the following formula [21]: 1 L of deionized water into which the next components at the mentioned concentrations (g/L) were dissolved (Xanthan gum (0.92), KCl (1.2), NaCl (0.85), MgCl2 (0.05), CaCl2 (0.13), NaH2PO4 (0.13), C8H8O3 (0.13)).
6. The pH of the tested erosive media was measured immediately before the insertion of the specimens in the acid-containing glass tubes, using a pH meter device (mPA—210P; MS Tecnopon Equipamentos especiais LTDA), before the insertion of the bars.

A 100 ml of each of the media was used to fill glass bottles into which the bars were inserted and then covered with lids. The bottles were kept in an incubator (Grant OLS 200, Grant Instruments Cambridge Ltd., Shepreth, UK) at 37 °C for 24 h under constant slow shaking of 70 rpm [22, 23]. This immersion time corresponds to 2.5 years of clinical exposure to these acids, which suggested that teeth are exposed to these acids three times/day with a duration of 30 s/exposure [24, 25]. All bars were then cleaned in distilled water for 10 min using ultrasonic cleaner and subsequently air-dried.

### Vickers microhardness (VHN)

The hardness of studied groups (n = 10/subgroup) was evaluated at room temperature using Vickers microhardness tester (FM-700, Future Tech Corp., Japan) with a load of 500 gf for 10 s. Five indentations were placed 0.5 mm apart from each other in a horizontal direction across the tested specimen (UNI EN ISO 6507) [26]. The two diagonal lines generated from each indentation were measured using the microhardness tester machine which automatically generates the VHN for the resulted indentations according to the following equation [27]:

$${\text{VHN}} = \frac{1.8544 \times P}{{d^{2} }}$$

where Hv is the Vickers hardness number in kg/mm^2^, P is the indenter load in kg and d is the diagonal length of the impression in mm.

The average of 5 VHN readings was counted as one for each specimen. According to the Standard Test Method for Vickers Indentation Hardness of Advanced Ceramics ASTM, any resulted indentation, shows ragged chipped or spalled edges, asymmetric diagonal lines, and a shift in the location of the tip of the indentation was excluded.

### Quantitative fractographic analysis and fracture toughness (KIC)

**Fig. 1:**
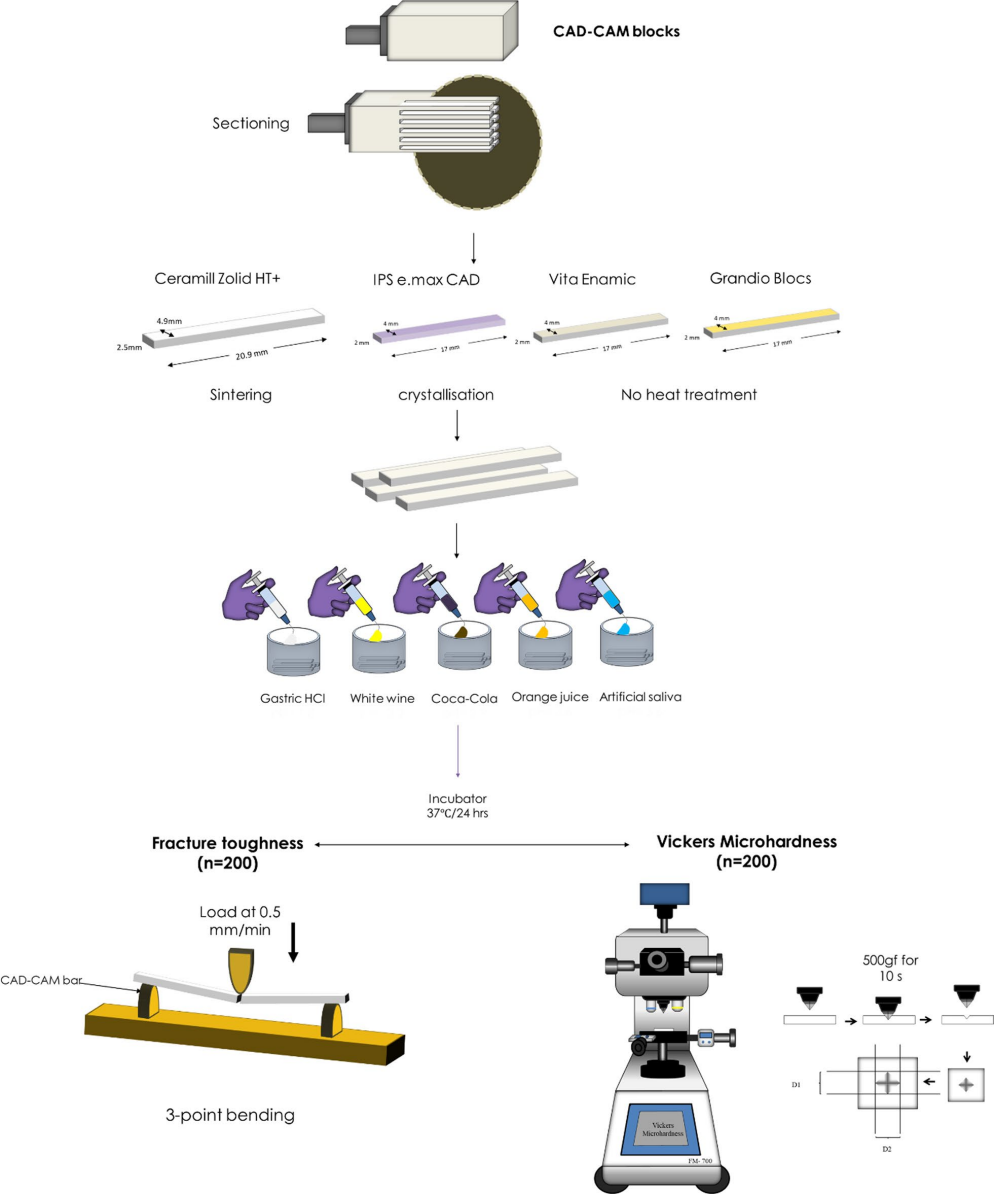
Schematic diagram showing the flow of specimens’ preparation and the tests employed. One hundred bard from each CAD-CAM material type were sectioned. Zirconia and lithium disilicate bars underwent sintering and crystallisation firing cycles, respectively, while nanohybrid resin composite and polymer-infiltrated glass network ceramic did not receive any firing treatment. All bars were embedded in 4 erosive media and artificial saliva for 24 h at 37℃. Half of the bars were then tested for microhardness and the other was tested for fracture toughness through quantitative fractographic method

Tested bars from each material group (n = 10/subgroup) underwent a 3-point flexural strength testing using a universal testing machine (Tinius Olsen model no 5ST, Surrey, UK). A brass-metal custom-made device [28] was made to mount each bar so that the span length was 14 mm following the International Organization for Standardization guidelines (ISO 6872: 2008) [22]. A load cell capacity of 5000 N loaded the central part of mounted bars at a crosshead speed of 0.5 mm/min till fracture [29] (Fig. 1). The fractured surfaces were gold palladium-sputtered and examined under the SEM (JEOL, JSM- IT200, Japan) at 60× magnification to examine the origin and pattern of the fracture.

Fracture toughness (K_IC_) can be calculated through the quantitative fractographic analysis method [30]. In this approach, surface fracture-initiating flaws are identified, and their sizes are calculated under SEM. Based on the size of the surface flaw that initiated the fracture, fracture toughness can be calculated according to Eqs. (1) and (2) [31].

1 $${\text{K}}_{{{\text{IC}}}} = {\text{Y}}.\sigma f.\sqrt c$$

where K_IC_ is the fracture toughness in MPa m^1/2^, Y is the geometry constant = 1.24 [32]. $\sigma f$ is the flexural strength in MPa and c is the fracture-initiating flaw size in µm.

Fracture initiating flaw, also known as critical defect size, can be obtained by the following equation

2 $${\text{c}} = (ab)^{1/2}$$

where c is the critical defect size in µm *a* is the height of the defect origin and *b* is its half-width in µm.

### Statistical analysis

Statistical analysis was performed using statistical software (SPSS Inc version 22, Chicago, IL, USA). The normality of data obtained was checked for all the variables using the Shapiro–Wilk test. All variables showed normal distribution, so mean and standard deviation (SD) were calculated, and parametric analysis was adopted. Two-way analysis of variance (ANOVA) was used to compare the effect of different erosive media and different CAD-CAM materials as two independent variables on surface microhardness and fracture toughness of CAD-CAM materials.

## Results

Simulated gastric HCl pH was found to be 1.2, while the white wine, orange juice, Coca-Cola®, and artificial saliva were found to be 3.3, 2.7, 3.9, and 7, respectively.

### Vickers microhardness and fracture toughness

Descriptive statistics of mean Vickers microhardness number and fracture toughness are presented in Table 1. Two-way ANOVA results showed that each of the independent variables (CAD-CAM material and erosive media) or their interaction had a statistically significant effect (*P* = 0.00) on the measured Vickers microhardness number and fracture toughness. The greatest influence was for the material (partial eta squared η_P_^2^ = 0.999 and 0.989 for VHN and K_IC_, respectively) followed by the interaction effect (η_P_^2^ = 0.624 and 0.782 for VHN and K_IC_, respectively) while erosive media had the lowest effect (η_P_^2^ = 0.251 and 0.322). Generally, the highest VHN was found in Ceramill Zolid HT+ followed by statistically significantly lower VHN in IPS e.max CAD, and Grandio Blocs, while Vita Enamic showed the lowest significant VHN regardless of the erosive media. In Ceramill Zolid HT+ group, zirconia _saliva_ showed the highest statistically significant (*P* = 0.00) microhardness number compared to the rest of the groups (1342.9 ± 6.9). No significant difference was found in microhardness between zirconia _HCl_ and zirconia _orange_ groups. Significantly higher VHN were found in zirconia _wine_ and zirconia _Coca-Cola®_. In IPS e.max CAD groups, erosive media did not significantly influence the VHN of the specimens. In Vita Enamic groups, HCl erosive media significantly deteriorated the microhardness of the material compared to the rest of the media, while all media had no significant influence on the VHN in all Grandio Blocs groups.

**Table 1:** Mean ± standard deviation of microhardness (VHN) and fracture toughness (K_IC_) of studied CAD-CAM materials among different erosive media

Materials	Media	Vickers microhardness (VHN)	K_Ic_ MPa m^1/2^
Ceramill Zolid HT+	Artificial saliva	1342.9 ± 6.9^A^	3.98 ± 0.21^A^
	HCl	1252.5 ± 2.5^B^	3.64 ± 0.33^A^
	White wine	1299.3 ± 7.2^C^	3.72 ± 0.29^A^
	Coca-Cola®	1283.4 ± 11.7^C^	3.76 ± 0.11^A^
	Orange	1248.7 ± 2.9^B^	3.81 ± 0.37^A^
IPS e.max CAD	Artificial saliva	606.9 ± 2.9^D^	1.89 ± 0.23^B^
	HCl	603.9 ± 4.3^D^	1.34 ± 0.34^C^
	White wine	588.9 ± 5.9^D^	1.62 ± 0.29^B^
	Coca-Cola®	601.7 ± 2.4^D^	1.77 ± 0.31^B^
	Orange	603.2 ± 2.1^D^	1.81 ± 0.19^B^
Vita Enamic	Artificial saliva	181.6 ± 2.7^E^	0.76 ± 0.17^D^
	HCl	155.5 ± 4.3^F^	0.63 ± 0.21^E,F^
	White wine	175.9 ± 4.6^E,F^	0.68 ± 0.19^D,F^
	Coca-Cola®	164.9 ± 2.9^E,F^	0.65 ± 0.18^F^
	Orange	163.1 ± 13.1^E,F^	0.64 ± 0.17^F^
Grandio Blocs	Artificial saliva	109.3 ± 2.5^G^	1.21 ± 0.09^C,G^
	HCl	103.4 ± 1.6^G^	1.03 ± 0.13^G^
	White wine	101.5 ± 1.7^G^	1.09 ± 0.11^G^
	Coca-Cola®	105.4 ± 1.8^G^	1.13 ± 0.2^G^
	Orange	99.6 ± 2.9^G^	1.01 ± 0.08^G^

No statistical significance (*P* > 0.05) is indicated by the same superscript capital letter in columns when comparing different materials in different media and by the same superscript numbers when comparing initial versus residual mean 3-point flexural strength

Fracture toughness was the highest in Ceramill Zolid HT+ groups followed by IPS e.max CAD and Grandio Blocs, while the Vita Enamic groups showed the lowest values regardless of the erosive media used. Erosive media did not significantly change the fracture toughness of zirconia. Similarly, for Grandio Blocs and IPS e.max CAD except for HCl-immersed specimens in the IPS e.max CAD group; a significant reduction in K_IC_ was found (*P* = 0.03). HCl had the highest significant impact on the K_IC_ of Vita Enamic specimens followed by orange juice and Coca-Cola® while white wine showed the least effect.

### Fractographic analysis

**Fig. 2:**
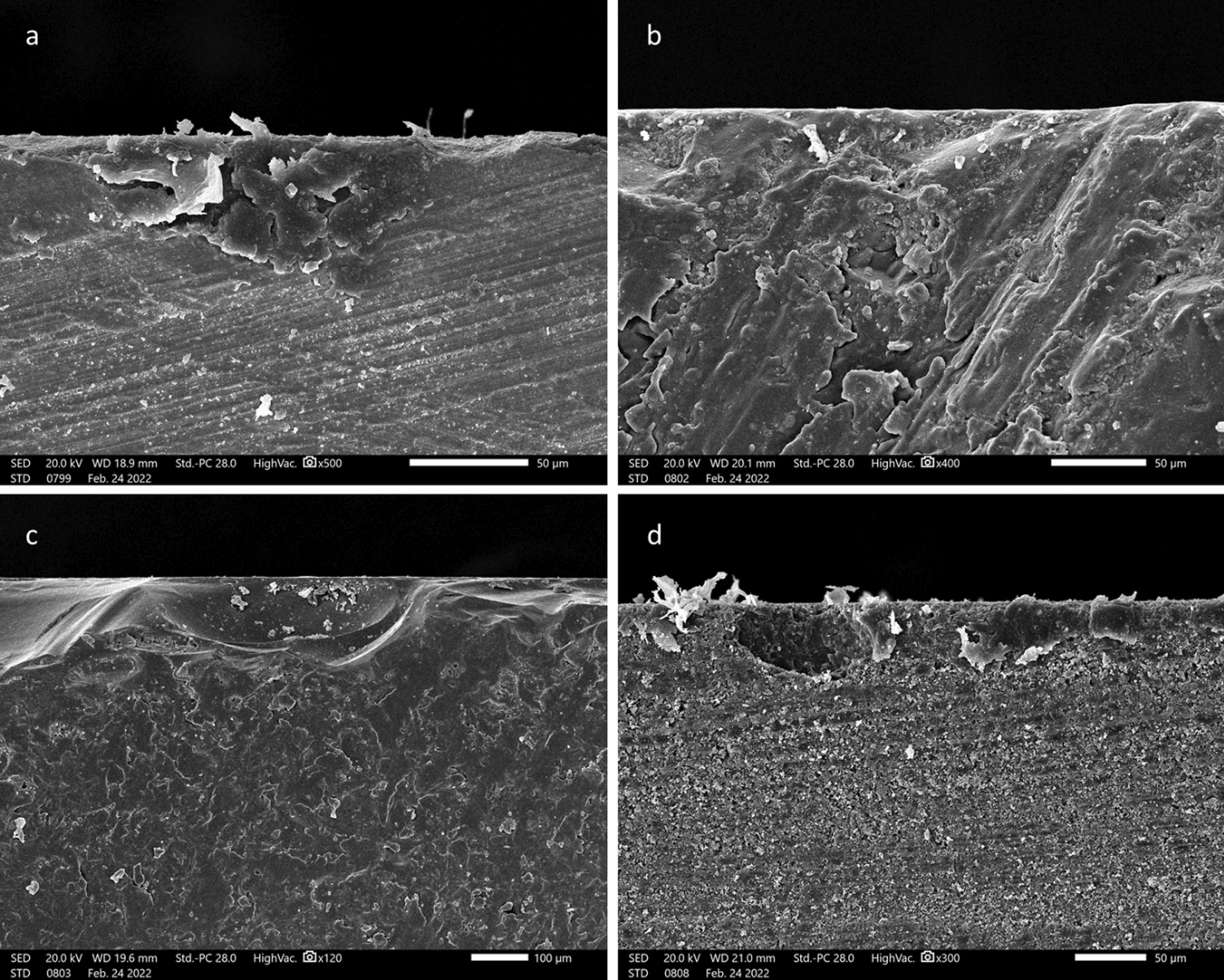
SEM images showing the critical size defects for groups: **A** Ceramill Zolid HT+, **B** IPS e.max CAD, **C** Vita Enamic, and **D** Grandio Blocs. Hackle lines originate from the surface defects indicating the direction of fracture

Scanning electron microscope images of the fractured surfaces of CAD-CAM specimens showed the largest defect surface flaws on Vita Enamic (187–210 µm) followed by Grandio Blocs and IPS e.max CAD (89–113 µm and 75–92 µm, respectively) while zirconia specimens showed the smallest defect sizes (36–41 µm) (Fig. 2). All fractures originated from surface flaws at the surface of the material with hackle lines originating from the defect sizes indicating the pattern and direction of fracture.

## Discussion

The current study aimed to investigate the effect of different erosive media on microhardness and fracture toughness of monolithic zirconia, lithium disilicate, a PICN, and a nanohybrid resin composite. A statistical significance between the studied groups was found and, therefore the null hypothesis was rejected.

There was no clear and standardized protocol to precisely simulate the erosive effect of acids of the in-vivo condition to be applied to an in-vitro model. Citric acid at a concentration of 4% and immersion time of 16 h at 80 °C has been the recommended protocol according to ISO standards for testing the solubility of dental ceramics [33]. However, the present study followed the erosive episodes that patients with GERD might be subjected to and applied for all employed acids [24, 25].

The effect of acid-containing media on CAD-CAM restorations has been studied in the literature in terms of whether different erosive media at different pH values might affect optical properties [3, 34], microhardness [20, 35], and mechanical properties [22]. In the literature, the simulated gastric HCl has been the most erosive to dental enamel and dentin compared to acid-containing food and drinks [6, 22, 36]. The pH of the gastric acid (1.2–1.5) is significantly below the limit (pH = 5.5) at which dental hard tissues start to dissolve [2]. It has been reported that gastric HCl can dissolve glass-based ceramic materials [37] and indirect resin composites [2]. Other studies reported no significant effect of erosive acids on CAD-CAM materials [38, 39]. This diversity of the outcome can be explained by the different materials used, different immersion times in erosive media, and different methodological designs employed.

The growing trend of biomimetics and bioemulation concept in aesthetic dentistry has been accompanied by the higher use of all ceramic and tooth-coloured restorations [40]. Patients now are more aware of the importance of keeping a natural beautiful smile and have become more well-educated about the recent technologies in dentistry that focus on restoring both of aesthetics and function. The CAD-CAM dental materials used in the study were examples of esthetic restorations which can mimic the beauty of natural teeth with satisfying mechanical properties. Monolithic zirconia, lithium disilicate, hybrid ceramics and nano-hybrid resin composites have been used extensively in the literature as potential dental materials that can provide the targeted optical and mechanical properties [41]. Patients with dental erosion who need esthetic restorations to their eroded teeth might request one of these dental material categories. However, the effect of erosive environment on these materials were questionable. It was a paramount importance to test how these materials would interact with the erosive media. The exposure of these materials to erosive acids is questionable as surface deterioration might be resulted and hence doubt their mechanical properties [3]. The effect of acid-containing beverages on lithium disilicate has been studied in the work of Flavia and co-authors [4]. They reported a significant reduction in the microhardness of the material secondary to the exposure to wine and Coca-Cola®. Acids might cause a disruption to the silica phase of lithium disilicate through leaching out of alkaline ions such as Al, Si, and Zr [4]. CAD-CAM resin composites outweigh the direct resin composites in many aspects. They were found to have higher mechanical and physical properties owing to their nature of manufacturing [42]. They are processed under isostatics conditions producing homogenous material with optimal mechanical properties comparable to glass–ceramics [42]. Due to their excellent mechanical properties, their application in restorative dentistry has been extended to involve single crowns, onlays, and overlays. PICN is a category of hybrid ceramics released to the market by the manufacturers that combine the advantages of resin composites and mechanical properties of glass–ceramics [43]. In the current study, erosive acids significantly deteriorated the VHN and fracture toughness of the material. Similar findings were reported by Şen and co-authors [44] and Sagsoz et al. [45].

The surface microhardness of the material indicates how hard a material is so that it resists the indentation. The higher the microhardness of the material, the higher mechanical properties shall be expected. In the current study, a significant difference in VHN was found between different CAD-CAM materials. CAD-CAM materials have been extensively tested for microhardness in the literature [46–48]. Zirconia specimens were the hardest followed by lithium disilicate and resin composites, while the PICN was the softest. The exposure of different erosive media significantly reduced the microhardness of zirconia and Vita Enamic specimens, while no effect was detected for the lithium disilicate and nanohybrid composite. This finding is not consistent with previous work [44]. The authors subjected the PICN to 75% ethanol. Ethanol at this concentration has been reported to have a degrading effect to the interface between inorganic fillers and organic resin matrix leading to dissolving of the material and hence, microhardness was significantly decreased [44]. However, the ethanol-containing white wine used contains only 12.5% ethanol which is still way below causing any dissolving effect.

Grandio Blocs showed higher microhardness values compared to the PICN (Vita Enamic), in agreement with a previous study [20]. Grandio Blocs, in the current study, had a high resistance to the erosive effect of different acids. This finding contradicts the study of Trussi and co-authors [49] and Marcela et al. [50]. They reported that acidic exposures might cause hydrolysis of methacrylate ester bonds leading to degradation of the polymer matrix of resin composites. Exposure of resin composites to acids might increase the water sorption of the material with a subsequent resin matrix expansion and creation of spaces between the molecules within the material. This could lead to the leaching out of the inorganic fillers and overall degradation of the material [51, 52]. This contradiction could possibly be explained by the solid microstructure of Grandio Blocs containing BIS-GMA and TEGDMA with significant polymer cross-linking within the material.

Few studies investigated the effect of gastric HCl on hybrid ceramics [22, 35]. The simulated gastric HCl has significantly decreased the microhardness of Vita Enamic compared to IPS e.max CAD, in consistency with previous work [3]. The significant difference in the microhardness between both materials could be attributed to the material type rather than the effect of acids. IPS e.max CAD has been reported to be a significantly harder material compared to Vita Enamic [53].

The continuous development of zirconia material has enabled its use as a whole restoration rather than being a core substructure for all-ceramic restorations [6]. Monolithic zirconia has been attracted by clinicians as a promising dental material to be used whenever both function and esthetics are the main objectives. The effect of acids on monolithic zirconia has been studied in a previous work of Althobity et al. [54]. The authors reported no significant change to zirconia microhardness after exposure to carbonated and citric acids. However, in the present study, orange juice which is the main source of citric acid significantly decreased the microhardness of zirconia. Citric acid ions in orange juice can form bonds with the metal oxides of zirconia leading to leaching out of surface crystals and hence, degradation of zirconia [55].

Fracture toughness can be defined as the material’s resistance to crack propagation [56]. It is one of the clinically relevant mechanical properties to be measured as it reflects how the material might behave in the clinical situation. Fracture is considered to be on top of the most frequent failures of dental restorations in dental practice [57]. Thus, it was important to investigate how the erosive media might affect the materials’ ability to resist the propagation of any possible surface flaws induced by the erosive acids. The single-edge notch method has been proposed in the literature to assess the fracture toughness of dental materials. In these methods, an intentional crack, in form of a v-shaped notch, is created at the edge of a bar-shaped specimen of the tested material. The specimen is then loaded so that the v-shaped notch is at the tension surface of the specimen in a three-point flexure testing. Other artificially-created surface flaws such as Vickers [58] or Knoop micro-indentations were used to measure fracture toughness [59, 60]. The surface crack on flexure method (SCF) has become the ASTM standard for measuring fracture toughness of brittle ceramics. In this approach, fracture toughness is calculated by measuring the resulted cracks from an indented surface at the fracture site [61]. However, failure of specimens due to large artificially created notches or indentations does not reflect the real clinical situation. In practice, failure of dental restorations might originate from surface micro-flaws that propagate under intermittent occlusal forces leading to fracture. These microflaws act as stress concentrators which when subjected to enough occlusal stresses, microcracks are consequently initiated and propagate to fail the restoration [18, 56]. To simulate the clinical condition, the fracture toughness was measured through the quantitative fractographic analysis method as it relies on measuring the natural flaws that caused the failure of specimens [18]. This approach has been extensively employed in the literature as a reliable method to measure fracture toughness of dental materials [18, 28, 56, 58].

To the authors’ knowledge, there are no previous studies that investigated the effect of erosive media on different CAD-CAM materials. Zirconia showed the highest resistance to the propagation of acid-induced surface defects compared to lithium disilicate and other CAD-CAM materials used. The effect of simulated gastric acid HCl on zirconia has been previously studied and no significant changes in the material mechanical properties were reported [34], in agreement with the findings of the present study. This could be possibly due to the crystalline nature of zirconia and lack of glassy phase and alkaline ions which could have leached out during the exposure to the acids leading to more surface deterioration [6]. Apart from simulated gastric HCl, erosive acids did not significantly deteriorate the fracture toughness of lithium disilicate glass–ceramic (IPS e.max CAD). Acid-exposed lithium disilicate specimens still showed higher fracture toughness values than the hybrid ceramic (Vita Enamic) and the nanohybrid resin composite (Grandio Blocs). This could be attributed to the high-volume content of lithium disilicate crystals (≈70%) which interlocks with the glassy matrix giving the material superior mechanical properties.

The current study is an in-vitro model to simulate an erosive exposure and its effect on the microhardness and fracture toughness of four CAD-CAM dental materials. This simulation may not accurately reflect the erosive environment in the clinical situation in which other factors might affect the severity of the erosive acids such as the buffering capacity of saliva. Therefore, the results of this study are only applicable to the tested conditions. Further long-term in-vivo studies are recommended to draw a clearer image of the CAD-CAM materials’ survival in different erosive media.

## Conclusion

Based on the findings of this in-vitro study, the following conclusions were drawn:

1. Zirconia had the highest VHN and K_IC_ values followed by IPS e.max CAD, Grandio Blocs, while Vita Enamic scored the lowest significant values.
2. Except for Vita Enamic, different erosive media did not significantly affect the VHN and fracture toughness of zirconia, IPS e.max CAD and Grandio Blocs.

## Data Availability

The datasets used and/or analysed during the current study are available from the corresponding author on reasonable request.

## Abbreviations

CAD-CAM: Computer aided design- computer aided manufacturing
GERD: Gastro oesophageal reflux disease
HCl: Hydrochloric acid
ANOVA: Analysis of variance
